# High Peripheral Blood Th17 Percent Associated with Poor Lung Function in Cystic Fibrosis

**DOI:** 10.1371/journal.pone.0120912

**Published:** 2015-03-24

**Authors:** Emily M. Mulcahy, Jo B. Hudson, Sean A. Beggs, David W. Reid, Louise F. Roddam, Margaret A. Cooley

**Affiliations:** 1 School of Medicine, University of Tasmania, Hobart, Tasmania, Australia; 2 Royal Hobart Hospital, Hobart, Tasmania, Australia; MRC National Institute for Medical Research, UNITED KINGDOM

## Abstract

People with cystic fibrosis (CF) have been reported to make lung T cell responses that are biased towards T helper (Th) 2 or Th17. We hypothesized that CF-related T cell regulatory defects could be detected by analyzing CD4^+^ lymphocyte subsets in peripheral blood. Peripheral blood mononuclear cells from 42 CF patients (6 months–53 years old) and 78 healthy controls (2–61 years old) were analyzed for Th1 (IFN-γ^+^), Th2 (IL-4^+^), Th17 (IL-17^+^), Treg (FOXP3^+^), IL-10^+^ and TGF-β^+^ CD4^+^ cells. We observed higher proportions of Treg, IL-10^+^ and TGF-β^+^ CD4^+^ cells in CF adults (≥ 18 years old), but not children/adolescents, compared with controls. Within the CF group, high TGF-β^+^% was associated with chronic *Pseudomonas aeruginosa* lung infection (*p* < 0.006). We observed no significant differences between control and CF groups in the proportions of Th1, Th2 or Th17 cells, and no association within the CF group of any subset with sex, CFTR genotype, or clinical exacerbation. However, high Th17% was strongly associated with poor lung function (FEV1 % predicted) (*p* = 0.0008), and this association was strongest when both lung function testing and blood sampling were performed within one week. Our results are consistent with reports of CF as a Th17 disease and suggest that peripheral blood Th17 levels may be a surrogate marker of lung function in CF.

## Introduction

T cell-mediated immune responses in people with cystic fibrosis (CF) have been reported to be biased towards either T helper (Th)2- or Th17-dominated responses [1–14]. The high prevalence of Th2-mediated allergic bronchopulmonary aspergillosis [15] and the presence of a high number of Th17 cells and cytokines in airways or lung-draining lymph nodes from people with CF [13] support this concept. However, the extent to which these alterations are directly related to dysfunctional cystic fibrosis transmembrane conductance regulator (CFTR) is not clear, because elevated levels of Th17 cells have also been reported in the lungs of individuals with a range of other inflammatory lung disorders [16].

Recognition of antigen by T cells triggers activation and maturation of the T cells to effector cells of different types. An early step in this process requires the influx of Ca^++^ ions into the T cell, and the functional outcome (e.g. Th1/Th2/Th17) depends partly on the strength of this Ca^++^ flux. Because CFTR is primarily a chloride ion channel that indirectly modulates Na^+^ and Ca^++^ flux across the membrane, and has been reported to be expressed on lymphocytes [10,17], we hypothesized that altered ion transport resulting from defective CFTR alters T cell responses to antigenic stimulation, and that this alteration may manifest as systemic changes in the relative proportions of different CD4^+^ T cell subsets. In support of this, varying alterations in the proportions of FOXP3^+^ Treg [18], Th17 [19], mucosal-associated invariant T (MAIT) [20] cells and changes in the relative production of a number of cytokines in peripheral blood of people with CF have been reported, but it is unclear to what extent this is related to the underlying CFTR defect or whether it is related to infection.

We measured the proportions of three effector (Th1, Th2 and Th17), and three regulatory (Treg, IL-10^+^ and transforming growth factor (TGF)-β^+^) CD4^+^ subsets in the peripheral blood of people with CF, compared them with those in age-matched controls, and analyzed the CF group for associations of each subset with demographic and clinical variables.

## Materials and Methods

### Study participants and blood collection

This study was approved by the Tasmanian Human Research Ethics Committee (reference numbers H008013 and H0012530). Written informed consent was obtained from study participants or from the guardian of child participants < 18 years old. The CF group was recruited from those attending the CF outpatient clinics at the Royal Hobart Hospital (RHH), Tasmania, Australia, or who were admitted to RHH because of clinical exacerbations, and comprised 20 adults (9 men, 11 women) aged 18–54, and 22 children/adolescents (8 boys, 14 girls) aged 0–17. Information including CFTR genotype (if available), current antimicrobial therapy, clinical status (i.e., stable or exacerbating, exacerbation defined as requiring admission to hospital and administration of intravenous antibiotics [21]) and sputum microbiology at date of blood collection were recorded. Lung function testing results as forced expiratory volume in 1 sec, % predicted (FEV1% predicted), calculated as defined by the Australian Cystic Fibrosis Data Registry [22], were recorded if available. The dataset is available as S1 Dataset.

The control group comprised 51 adult volunteers (24 men, 27 women) aged 19–61 and 17 children/adolescents (9 boys, 8 girls) aged 2–17: these were healthy individuals with no history of chronic illness, no known family history of CF, and no recent (i.e., within the previous two weeks) history of infectious illness.

Peripheral venous blood was collected in lithium heparin and processed within 5 h of collection. Peripheral blood mononuclear cells (PBMC) were isolated and resuspended in Dulbecco’s phosphate-buffered saline (DPBS)/10% fetal bovine serum (FBS).

### Activation, staining and flow cytometric analysis of CD4^+^ cells

PBMC were activated and stained according to the manufacturer’s instructions (BD Biosciences, Mountainview, CA). Detailed methods are provided in the (S1 Methods). For identification of Th1 and Th2 cells, or Th17 and Treg cells, four-color antibody cocktails (Th1/Th2/Th17 phenotyping kit, Treg/Th17 phenotyping kit, BD Biosciences) were used. For identification of IL-10^+^ and TGFβ^+^ cells, anti-CD4 PerCP-Cy5.5, anti-TGFβ1 PE, and anti-IL-10 APC (all BD Biosciences) were used. Anti-CD4 PerCP-Cy5.5, PE-conjugated mouse IgG1κ and APC-conjugated mouse IgG2aκ (all BD Biosciences) were used as isotype control. All samples were analyzed on a CyAn ADP flow cytometer (Beckman Coulter, Lane Cove, NSW, Australia) using Summit software (version 4.3.2, Beckman Coulter). At least 100,000 gated events were collected for each sample. Postacquisition analysis was performed using FlowJo software (version 9.5.2, Tree Star, Ashland, OR). Representative gating and fluorescence histograms are shown in (S1 Fig.). CD4^+^ values were expressed as a percent of total lymphocytes; all other subsets as a percent of CD4^+^ cells.

### Statistical analysis

All statistical analysis was performed using Graphpad Prism V6 (Graphpad Software, La Jolla, CA). Because there are known interactions between CD4 T cell subsets (e.g., higher levels of Th1 imply lower levels of Th2), we performed individual comparisons for each subset, rather than multiple comparisons. Because most variables were not normally distributed, comparison of pairs of continuous variables and of continuous variables between each pair of groups was made using the Mann—Whitney *U* test. Analysis of correlations between two continuous variables was performed using Spearman’s test. All *p* values are two-tailed; *p* < 0.05 was regarded as significant.

## Results

### Analysis of CD4^+^ subset percentages

All the analysis of CD4^+^ T cells in this study was based on percentages (CD4^+^ as percent of lymphocytes, all other subsets as percent of CD4^+^ cells). We also evaluated the absolute numbers of these cells in those participants for whom differential counts were available: the CF group had low to normal absolute numbers of lymphocytes (0.4–4.5 × 10^9^/L) and CD4^+^ cells (250–1500 cells/mL), and consideration of absolute numbers increased the variability but did not alter the relationships (data not shown). For this reason, and because differences in proportion would be more likely to reflect intrinsic alterations in the “shape” of the T cell mediated immune response than would differences in absolute number, we performed all analyses using subset percentages.

### Comparison between control and CF groups

Table 1 summarizes the sex, median age, and CD4^+^ subset percentages in the control and CF groups. There were no significant differences between the groups in age or sex, the overall percent CD4^+^ cells, or the percentage of any of the effector (Th1, Th2 or Th17) subsets. However, all three regulatory CD4^+^ subsets (Treg, *p* = 0.016; IL-10^+^, *p* = 0.0001; and TGFβ^+^, *p* < 0.0001) were present at significantly higher percentages in the CF group than in controls. As a guide to whether the differences in regulatory T cell subsets were inherent to CF or developed in response to chronic inflammation/infection, we subdivided both control and CF groups into adults (over 17 years (n = 51 controls, n = 20 CF) and adolescents/children (17 years or under (n = 17 controls, n = 22 CF). The percentages of all three regulatory subsets remained significantly higher in the adult CF group than in adult controls, but there were no significant differences in any subset between control and CF adolescents/children (Table 2).

**Table 1:** Comparison of demographics and CD4^+^ T cell subsets in control and cystic fibrosis groups.

	Controls	Cystic Fibrosis	*P-*value (Mann—Whitney)
N	68	42	
Age median (range)	21 (2–61)	15 (0.5–53)	0.22
Sex male (%)	33 (48%)	20 (47%)	
CD4^+^% median (range)	48.8 (29.7–66.0)	46.8 (28.3–66.1)	0.48
Th1% median (range)	7.9 (3.0–22.4)	7.0 (1.2–24.6)	0.09
Th2% median (range)	2.3 (0.4–9.8)	1.7 (0.35–8.5)	0.15
Th1/Th2 ratio median (range)	4.5 (1.3–11.3)	4.8 (0.7–10.4)	0.81
Th17% median (range)	0.47 (0.08–2.26)	0.43 (0.03–1.98)	0.30
Treg% median (range)	5.8 (1.8–11.2)	7.1 (2.5–12.8)	**0.015**
Treg/Th17 ratio median (range)	11.9 (2–57)	16.2 (3.2–441)	0.066
IL-10^+^% median (range)	1.18 (0.35–3.32)	1.63 (0.41–4.16)	**0.0001**
TGFβ^+^% median (range)	0.027 (0.008–0.190)	0.055 (0.017–0.417)	**<0.0001**

*P*-values (Mann—Whitney) of significant differences are shown **bold.**

**Table 2:** Comparison of CD4^+^ T cell subset percentages in adults and children in control and CF groups.

	Adults			Children and adolescents		
	Control	CF	*P* value*	Control	CF	*P* value*
N	51	20		17	22	
Age median (range)	22 (18–61)	24 (18–53)	0.13	8 (2–17)	11 (0.5–17)	0.22
Sex male (%)	24 (47%)	12 (60%)		9 (51%)	8 (36%)	
CD4^+^% median (range)	50.4 (29.7–66.0)	47.4 (28.3–66.1)	0.18	42.1 (32.7–52.6)	46.8 (33.2–61)	0.15
Th1% median (range)	9.6 (3.0–22.4)	8.5 (3.5–24.6)	0.82	4.6 (3.0–9.6)	5.4 (1.2–10.7)	0.98
Th2% median (range)	2.5 (0.7–9.8)	2.3 (0.4–8.5)	0.74	1.4 (0.4–5.0)	1.2 (0.4–8.4)	> 0.99
Th1/Th2 median (range)	4.6 (1.3–11.3)	5.1 (1.0–10.4)	0.67	3.8 (1.3–8.5)	3.9 (0.7–8.8)	0.91
Th17% median (range)	0.49 (0.15–2.26)	0.52 (0.04–1.98)	0.77	0.37 (0.08–1.08)	0.36 (0.03–1.02)	0.79
Treg% median (range)	5.5 (1.8–10.5)	6.8 (4.2–11.4)	**0.008**	7.6 (3.5–11.2)	7.2 (2.5–12.8)	0.77
Treg/Th17	10.6 (2–37)	14.8 (4–441)	0.32	18.6 (6.9–57)	18.6 (3.2–305)	0.93
IL-10^+^% median (range)	1.15 (0.35–2.63)	1.77 (0.41–4.16)	**0.0004**	1.27 (0.70–3.32)	1.5 (0.66–2.85)	0.29
TGFβ^+^% median (range)	0.022 (0.008–0.19)	0.093 (0.018–0.417)	**< 0.0001**	0.044 (0.020–0.143)	0.043 (0.017–0.301)	0.86

*P-values (Mann—Whitney) of significant differences are shown **bold**

Because it has been reported that CF patients have Th2-biased or Th17-biased responses, we also calculated the Th1/Th2 ratio and Treg/Th17 ratios. There was no significant difference between the groups in Th1/Th2 ratio (*p* = 0.8). The Treg/Th17 ratio in the CF group was much more variable than that in controls, and trended towards being higher (*p* = 0.066) (Table 1), consistent with the slightly higher Treg% in the CF group.

### Relationships of subset percentages in the CF croup with clinical parameters

To assess whether the percentage of any CD4^+^ subset was associated with relevant clinical parameters, we analyzed these within the CF group (Table 3), and assessed the correlations of the percentage of each CD4^+^ subset with continuous variables (age, FEV1% predicted) and any differences between subgroups divided by sex (M = 20, F = 22), genotype (DF508 homozygous = 18, DF508 heterozygous = 21, other = 3). We also compared clinically stable (n = 35) with exacerbating (n = 7) patients, those with (n = 15) or without (n = 27) chronic *P*. *aeruginosa* infection (defined as the regular culture of *P*. *aeruginosa* from the sputum or respiratory secretions, on two or more occasions, extending over 6 months or a shorter period [23]), with (n = 16) or without (n = 26) current *Staphylococcus aureus* infection, with (n = 7) or without (n = 35) current *Aspergillus fumigatus* infection (both assessed on sputum microbiology), and with (n = 21) or without (n = 21) current treatment with any antimicrobial. Of note, only 3/7 individuals with identified *A*. *fumigatus* infection had diagnosed allergic bronchopulmonary aspergillosis (ABPA).

**Table 3:** Associations of CD4^+^ subset percentages with demographic and clinical variables in the CF group.

Parameter [no. of patients]	CD4%	Th1%	Th2%	Th17%	Treg%	IL-10^+^%	TGFβ^+^	Th1/Th2	Treg/Th17
Age [42] (Spearman r)	–0.032	0.56	0.44	0.23	0.06	0.12	0.29	0.16	–0.11
*P*-value*	[0.84]	**[*0.002*]**	**[*0.018*]**	[0.16]	[0.73]	[0.46]	[0.07]	[0.43]	[0.51]
FEV1% pred. [32] (Spearman r)	–0.01	–0.45	–0.31	**–0.56**	**–0.42**	0.003	0.04	–0.25	–0.12
*P*-value	[0.95]	[0.06]	[0.19]	**[*0.0008*]**	**[*0.016*]**	[0.99]	[0.82]	[0.28]	[0.57]
FEV1% pred. stable patients only [25] (Spearman r)	–0.13	–0.45	–0.31	**–0.58**	–0.31	0.16	0.16	–0.10	–0.14
*P*-value	[0.52]	[0.06]	[0.19]	**[*0.0017*]**	[0.12]	[0.44]	[0.44]	[0.68]	[0.52]
**Median percent for each subset**									
Sex									
Male	46.3	7.8	1.80	0.43	7.1	1.60	0.090	4.8	14.4
Female	48.2	5.7	1.42	0.40	6.9	1.67	0.055	4.7	17.0
*P*-value	[0.25]	[0.38]	[0.32]	[0.49]	[0.96]	[0.69]	[0.13]	[0.84	[0.30]
Genotype									
DF508 homozygous [18]	47.8	5.7	1.55	0.41	6.7	1.76	0.053	4.9	13.3
DF508 heterozygous/other [24]	46.8	6.2	1.42	0.43	7.6	1.47	0.069	4.8	19.5
*P-value*	[0.74]	[0.85]	[0.99]	[0.69]	[0.12]	[0.48]	[0.66]	[1.0]	[0.28]
Stable [35]	47.0	6.2	1.42	0.43	7.0	1.65	0.053	4.7	17.0
Exacerbating [7]	48.2	13.1	2.35	0.65	7.1	1.56	0.079	5.90	9.9
*P*-value	[0.94]	[0.26]	[0.32]	[0.14]	[0.60]	[0.99]	[0.76]	[0.13]	[0.18]
Chronic *P*. *aeruginosa* [15]	46.6	9.6	2.32	0.66	6.8	1.44	0.095	5.8	11.2
No chronic *P*. *aeruginosa* [27]	47.8	5.7	1.63	0.36	7.3	1.63	0.041	3.9	18.7
*P*-value	[0.42]	[0.17]	[0.42]	[0.09]	[1.0]	[0.69]	***[0.006]***	***[0.04]***	[0.18]
Current *S*. *aureus* infection [16]	48.1	5.7	2.11	0.42	6.8	1.77	0.044	4.8	16.6
No *S*. *aureus* infection [26]	46.5	6.4	1.42	0.51	7.1	1.63	0.088	4.7	13.8
*P*-value	[0.59]	[0.79]	[0.50]	[0.51]	[0.76]	[0.53]	[0.17]	[0.87]	[0.34]
Current *A*. *fumigatus* infection [7]	46.3	8.9	1.80	0.43	6.3	1.54	0.091	4.9	18.8
No *A*. *fumigatus* infection [35]	47.5	5.4	1.52	0.43	7.1	1.65	0.048	4.4	15.8
*P*-value	[0.42]	[0.06]	[0.36]	[0.92]	[0.48]	[0.75]	[0.25]	[0.56]	[0.97]
Any current antimicrobial [21])	47.6	8.5	2.0	0.71	6.8	1.76	0.092	5.2	9.3
No current antimicrobial (21]	46.6	5.7	1.69	0.34	7.1	1.54	0.041	3.7	18.8
*P*-value	[0.68]	[0.07]	[0.35]	***[0.013]***	[0.93]	[0.42]	***[0.047]***	[0.13]	***[0.02]***

*P-values indicating significant differences are shown in **bold.**

To examine the effects of CFTR genotype, we divided the CF group into two subgroups: the first included heterozygous DF508 and other genotypes (n = 24), because only three of the CF group did not have at least one DF508 allele, and the second group comprised individuals homozygous for DF508 (n = 18). There was no significant difference detected in the percentage of any CD4^+^ T cell subset based on CFTR genotype.

To examine the effects of clinical status, we divided the CF group into clinically stable (n = 35) and exacerbating (n = 7) subgroups. There was no significant difference in any CD4^+^ subset between those who were clinically stable and those who were exacerbating.

With respect to lung infection, there was a significantly higher TGFβ^+^% (*p* = 0.006) and a trend towards a higher Th17% (*p* = 0.09) in participants with chronic *P*. *aeruginosa* infection. For those on current antimicrobial treatment (n = 21) the increases in both TGFβ^+^% and Th17% were significant (TGFβ^+^% *p* = 0.05; Th17% *p* = 0.01). However, no differences were detectable between participants with and without other infections, including *S*. *aureus* and *A*. *fumigatus*.

**Fig 1:**
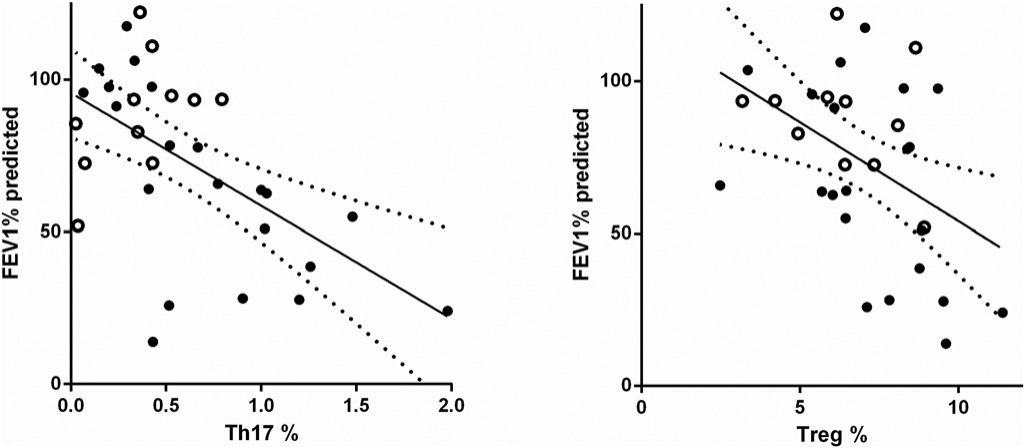
Correlation of Th17% and Treg % with FEV1% predicted. Analysis was performed using Spearman’s correlation. Individual results are shown; the linear regression line is shown as a solid line and the 95% confidence intervals as dotted lines. Subjects for whom lung function testing was performed within 2 weeks of blood sampling (n = 21): black circles; subjects for whom lung function testing was performed more than 2 weeks from blood sampling (n = 11): open circles.

Analysis of the relationship between CD4^+^ subset percentages and FEV1% predicted was performed on a subset of CF patients (n = 32; 17 adults, 14 children) for whom lung function testing results were available. This subgroup was slightly older (median age 19.5 years; included no children under 5 years) and had a higher proportion (59%) of males, but for other variables was representative of the whole cohort. With this subgroup, the correlation between Th17% and FEV1% predicted was extremely strong (*r* = –0.56, *p* = 0.0008, Spearman), and that with Treg% significant (*r* = –0.42, *p* = 0.016), with increasing percentages of both subsets being associated with poorer lung function (Fig. 1). Because lung function can change rapidly with treatment of clinical exacerbations, and not all lung function testing was performed on the same day as blood sampling, we also analyzed this association (a) after excluding all FEV1% samples from patients exacerbating on the day of blood sampling (n = 5) and (b) depending on the relative timing of blood sampling and lung function testing. When considering all clinically stable patients, the association with FEV% of Th17% remained (*r* = –0.58, *p* = 0.0017), whereas that with Treg% was no longer significant (*p* = 0.11) (Table 4). When data were analyzed by grouping patients depending on the time interval between blood sampling and lung function testing (Table 4), it became clear that the correlation was strongest when both tests were done within a week (*r* = –0.86, *p* < 0.0001 for Th17%; *r* = –0.62, *p* = 0.01 for Treg%, n = 16), and was not significant if lung function testing and blood sampling were separated by more than 2 weeks. We also compared the groups with and without antimicrobial treatment, but this was confounded because most of the patients on antimicrobials were adult and most not on antimicrobials were children (median age on antimicrobials 22 years, median age not on antimicrobials 13 years), and the groups also differed in median lung function, with most of those not on antimicrobials having very good lung function (median FEV 1% on antimicrobials 54%, median FEV 1% no antimicrobials 97%). However, the correlation of Th17% with FEV 1% was maintained within the group on antimicrobials (*p* = 0.03, n = 18).

**Table 4:** Correlation of Th17% with FEV1% predicted depending on time between lung function testing and blood sampling.

Group (no.)	Age years median (range)	% male	Th17% Spearman *r* (*p*-value)*	Treg% Spearman *r* (*p*-value)
All (32)	19.5 (6–53)	59%	–0.56 **(0.0008)**	–0.42 **(0.016)**
FEV1% predicted test on same day as blood sampling (10)	22 (10–53)	70%	–0.85 **(0.003)**	–0.60 (0.07)
FEV1% predicted test within 1 week of blood sampling (16)	22 (10–53)	63%	–0.86 **(< 0.0001)**	–0.62 **(0.01)**
FEV1% predicted test within 2 weeks of blood sampling (21)	20 (10–53)	67%	–0.72 **(0.0003)**	–0.49 **(0.02)**
FEV1% predicted test more than 2 weeks from blood sampling (11)	11 (6–38)	45%	0.49 (0.12)	–0.35 (0.29)

*P-values indicating significant differences are shown in **bold.**

## Discussion

This is, to our knowledge, the first demonstration that peripheral blood, rather than lung or bronchoalveolar lavage, Th17% is strongly inversely correlated with lung function (FEV1% predicted) in people with CF. A similar finding has been reported for people with chronic obstructive pulmonary disease (COPD), although in that study, the overall Th17% was also higher in COPD patients than in controls [24]. There is consensus that Th17-mediated inflammation in the lungs is an important part of the pathology of CF [11]. Although we were unable to detect any differences in the peripheral blood Th17% between controls and people with CF, the strong association within the CF group of high peripheral blood Th17% with poor lung function is consistent with an important association of Th17 cells with lung damage in CF [13,14,16]. It is striking that this strong correlation was detectable using peripheral blood Th17%, rather than, as in previous reports for CF, lung (bronchoalveolar lavage) Th17 levels [13,14], and was strongest in when lung function testing was performed within a week of blood sampling. In adults with CF, we do see a trend (*p* = 0.09) towards an association of high Th17% with chronic *P*. *aeruginosa* infection, and it has been reported that a Th17-biased lung immune response is a risk factor for acquisition of chronic *P*. *aeruginosa* infection, possibly because it is ineffective against that pathogen [14], so it is possible that the strong inverse correlation of peripheral blood Th17% with lung function may be at least partly related to a predisposition of those with high Th17% to acquire lung damage as the result of *P*. *aeruginosa* infection. However, we were not able to confirm this because of the relatively small number of patients with *P*. *aeruginosa* infection. This is also consistent with the association we observed in those being treated with antimicrobial therapy.

We also observed an association of Treg% with FEV1% predicted in the overall CF group, but when participants who were clinically exacerbating were excluded, this was not significant, supporting the concept that higher Treg% in adults with CF is related to infection/inflammation. It is interesting that high percentages of both Th17 and Treg were associated with very poor lung function, as these are often considered to be mutually antagonistic subsets [25], and it might have been expected that high Treg% would be associated with less inflammation and less lung damage. However, one possible explanation of this is the production of IL-17 by dysregulated Tregs that has been reported in COPD [24] and inflammatory bowel diseases [26].

The inverse correlation of Th17% in peripheral blood with FEV1% predicted could be related to a number of possible factors. First, it should also be noted that decreasing lung function in the CF group is also correlated with increasing age, but as we found no significant association of Th17% with age (Table 3), the association between Th17% and FEV1% predicted is unlikely to be related to age. Second, it may be that high levels of Th17 cells in the blood reflect very high levels of Th17 cells in the lung, which have been linked with lung damage [11], although no information is available about the relative homing to lung and periphery of Th17 cells. Third, it could be that, as has been reported for lung Th17 levels [14] high systemic Th17% predisposes to the acquisition of chronic *P*. *aeruginosa* infection, and that the infection causes the lung damage. Fourth, high peripheral Th17% may relate to an individual’s inherent propensity for bias towards Th17-dominated responses, which, like the propensity to Th2 allergic responses, is probably formed and detectable very early in life [27–29]; in this scenario, peripheral blood Th17% may be useful as a prognostic indicator of the likelihood of *P*. *aeruginosa* infection and rapid lung function decline in CF. However, as the correlation between Th17% and FEV1% predicted was strongest when lung function testing and blood sampling were closely linked in time, it seems more likely that this varies in concert with lung function, rather than predicting lung function decline. Further studies are required to confirm whether Th17% in blood has any predictive value for lung function decline.

In contrast to previous reports suggesting a Th17 and/or Th2 bias in CF T cell responses in lungs [13,14] or of a Th17 bias in peripheral blood lymphocytes [12], we did not see in this study any significant increase in the Th17% or Th2% in the peripheral blood of the CF group compared with controls, although CF patients showed greater variation in Th17% than did controls. There was also no significant difference between the CF group and controls in Th1/Th2 ratio or Treg/Th17 ratio, although the latter was much more variable in CF, largely because of variable Th17%. Overall, we found no evidence of either a Th17 or a Th2 bias in the peripheral blood of CF patients.

However, we did see significantly higher percentages of three regulatory CD4^+^ subsets, FOXP3+, IL-10+, and TGF-β^+^CD4^+^ cells, in the CF group compared with controls, differences which were significant only in the adults (>18 years). The elevation of CD4+IL-10+ cells was somewhat unexpected, as previous reports have suggested reduced levels of IL-10 production in people with CF [30]. Unlike the changes in the other two subsets, the increase in FOXP3+ cells was only marginally significant. Indeed, previous reports have suggested reduced levels of Tregs in children with CF [18,19]. Our pediatric group showed no significant changes in any subset we measured, but they were also mostly without chronic infections or antimicrobial treatment. With respect to the changes in our adult CF population, elevations in several regulatory CD4^+^ subsets have been reported to occur in response to chronic inflammation and infection [25,31,32]. Because chronic inflammation is characteristic of CF, it is not surprising that the percentages of all three regulatory subsets examined were elevated, and this perhaps also explains why significant differences are seen only in adults, who have of course been exposed to chronic inflammation/infection for longer than children. Despite the slightly elevated Treg% present in the CF group and the lack of significant changes in Th17%, there was no significant difference between control and CF groups in Treg/Th17 ratio. This may be partly because of the nature of our analysis that defined Tregs as including all CD4^+^FOXP3^+^ cells. Many definitions of Tregs include not only CD4 and FOXP3 expression, but also expression of markers including CD25 and/or CD127 [33]. In addition, Tregs can be divided, based on expression of a range of specific surface markers, into naive (antigen inexperienced) or memory Tregs [34], and subsets that home to different tissues [5]. It is likely that in the context of the constant infections in people with CF, that the relative numbers of these Treg subsets would differ from those in healthy controls. We also did not examine the function of any CD4^+^ subset, other than their capacity to produce their signature cytokine in vitro after polyclonal stimulation. Therefore, it is possible that cells that have similar surface markers/cytokine expression may differ in their functional activity. For example, it has been shown that FOXP3+ cells induced as a result of inflammation differ functionally from those “natural” Tregs that are present in all individuals [32,35]. Therefore, a detailed analysis of naive versus memory Tregs and of Tregs homing to different tissues may be informative to clarify how the Tregs present in CF patients differ from those in controls, and whether particular Treg subsets are associated with lung function decline or other clinical parameters.

The elevation of TGFβ^+^% and the trend to increased Th17% in patients with chronic *P*. *aeruginosa* infection is interesting, especially as in our cohort such increases are not associated with other infections including *S*. *aureus* and *A*. *fumigatus*. This suggests that the increases may be related to the persistent and highly inflammatory nature of the response to *P*. *aeruginosa*, and it is possible that these markers could be useful to monitor/predict infection, as previously suggested by Tiringer et al [14]. However, we are not able to evaluate the predictive ability of these markers based on our current data.

The strengths of this study are the relatively large numbers and the very broad age range of both control and CF groups, which allow some analysis of the relationships of both age and clinical variables with the selected T cell subset markers. However, the study has several weaknesses. First, it is cross-sectional, so no evaluation of the predictive ability of any of the markers can be made. Second, lung function measurements were not available for all patients, and were not always performed on the same day as blood collection. Third, while the number of subjects is larger than that in many studies, some of the subgroups are small, which impaired our ability to analyze differences between them.

In conclusion, our study for the first time suggests that high peripheral blood Th17% in people with CF is associated with poor lung function, and that it may be a surrogate marker of lung function. This could be particularly useful in children less than six years of age, in whom standard spirometry is generally not possible. While various forms of infant and preschool pulmonary function tests exist, they tend to be invasive and there is insufficient evidence to incorporate them into routine clinical care at this time. However, most children with CF will have blood tests done at least once per year as part of their annual review, so the development of a blood test that is correlated with changes in lung function could be clinically useful.

The elevated CD4^+^TGFβ^+^% specifically associated with chronic *P*. *aeruginosa* infection could also be clinically useful for similar reasons. However, further studies are required to determine whether these parameters have any useful prognostic value for predicting the progression of CF-related disease.

## Supporting Information

S1 Dataset Deidentified patient information and results. (XLSX)

S1 Methods Supplementary Methods. (DOCX)

S1 Fig Representative histograms demonstrating the gating strategy for flow cytometric analysis. All samples were gated on lymphocytes (A) then single cells (B) for determination of CD4+ percent. For determination of CD4^+^ subset percentages, cells were gated on CD4^+^ (C) then subsequent IL-4 vs. IFNγ (D), IL-17 vs. FOXP3 (E) and TGF-β vs. IL-10 (F) histograms were analyzed.
(TIFF)

## References

[1] McDonald TV, Nghiem PT, Gardner P, Martens CL. Human lymphocytes transcribe the cystic fibrosis transmembrane conductance regulator gene and exhibit CF-defective cAMP-regulated chloride current. J Biol Chem. 1992;267: 3242–3248. 1371114

[2] Skov M, Poulsen LK, Koch C. Increased antigen-specific Th-2 response in allergic bronchopulmonary aspergillosis (ABPA) in patients with cystic fibrosis. Pediatr Pulmonol. 1999;27: 74–79. 10088929 10.1002/(sici)1099-0496(199902)27:2<74::aid-ppul2>3.0.co;2-l

[3] Moser C, Kjaergaard S, Pressler T, Kharazmi A, Koch C, Hoiby N.The immune response to chronic Pseudomonas aeruginosa lung infection in cystic fibrosis patients is predominantly of the Th2 type. Apmis. 2000;108: 329–335. 10937769 10.1034/j.1600-0463.2000.d01-64.x

[4] Moss RB, Hsu YP, Olds L. Cytokine dysregulation in activated cystic fibrosis (CF) peripheral lymphocytes. Clin Exp Immunol. 2000;120: 518–525. 10844532 10.1046/j.1365-2249.2000.01232.x PMC1905557

[5] Hausler M, Schweizer K, Biesterfel S, Opladen T, Heimann G. Peripheral decrease and pulmonary homing of CD4+CD45RO+ helper memory T cells in cystic fibrosis. Respir Med. 2002;96: 87–94. 11860174 10.1053/rmed.2001.1217

[6] Brazova J, Sediva A, Pospisilova D, Vavrova V, Pohunek P, Macek M, et al Differential cytokine profile in children with cystic fibrosis. Clin Immunol. 2005;115: 210–215. 15885645 10.1016/j.clim.2005.01.013

[7] Allard JB, Poynter ME, Marr KA, Cohn L, Rincon M, Whittaker LA. Aspergillus fumigatus generates an enhanced Th2-biased immune response in mice with defective cystic fibrosis transmembrane conductance regulator. J Immunol. 2006;177: 5186–5194. 17015704 10.4049/jimmunol.177.8.5186

[8] Hartl D, Griese M, Kappler M, Zissel G, Reinhardt D, Rebhan C, et al Pulmonary T(H)2 response in Pseudomonas aeruginosa-infected patients with cystic fibrosis. J Allergy Clin Immunol. 2006;117: 204–211. 16387607 10.1016/j.jaci.2005.09.023

[9] Dubin P, McAllister F, Kolls J. Is cystic fibrosis a TH17 disease? Inflammation Research. 2007;56: 221–227. 17607545 10.1007/s00011-007-6187-2

[10] Mueller C, Braag SA, Keeler A, Hodges C, Drumm M, Flotte TR. Lack of cystic fibrosis transmembrane conductance regulator in CD3+ lymphocytes leads to aberrant cytokine secretion and hyperinflammatory adaptive immune responses. Am J Respir Cell Mol Biol. 2011;44: 922–929. 10.1165/rcmb.2010-0224OC 20724552 PMC3135852

[11] Tan HL, Regamey N, Brown S, Bush A, Lloyd CM, Davies JC. The Th17 pathway in cystic fibrosis lung disease. Am J Respir Crit Care Med. 2011;184: 252–258. 10.1164/rccm.201102-0236OC 21474644 PMC3381840

[12] Kushwah R, Gagnon S, Sweezey NB. Intrinsic predisposition of naive cystic fibrosis T cells to differentiate towards a Th17 phenotype. Respir Res. 2013;14: 138 10.1186/1465-9921-14-138 24344776 PMC3890528

[13] Chan YR, Chen K, Duncan SR, Lathrop KL, Latoche JD, Logar AJ, et al Patients with cystic fibrosis have inducible IL-17+IL-22+ memory cells in lung draining lymph nodes. J Allergy Clin Immunol. 2013;131: 1117–1129, 1129 e1111–1115. 10.1016/j.jaci.2012.05.036 22795370 PMC3488163

[14] Tiringer K, Treis A, Fucik P, Gona M, Gruber S, Renner S, et al A Th17- and Th2-skewed cytokine profile in cystic fibrosis lungs represents a potential risk factor for Pseudomonas aeruginosa infection. Am J Respir Crit Care Med. 2013;187: 621–629. 10.1164/rccm.201206-1150OC 23306544

[15] Knutsen AP, Slavin RG. Allergic bronchopulmonary aspergillosis in asthma and cystic fibrosis. Clin Dev Immunol. 2011: 843763 10.1155/2011/843763 21603163 PMC3095475

[16] Way EE, Chen K, Kolls JK. Dysregulation in lung immunity—The protective and pathologic Th17 response in infection. Eur J Immunol. 2013;43: 3116–3124. 10.1002/eji.201343713 24130019 PMC3947216

[17] Tang XX, Fok KL, Chen H, Chan KS, Tsang LL, Rowlands DK, et al Lymphocyte CFTR promotes epithelial bicarbonate secretion for bacterial killing. J Cell Physiol. 2012;227: 3887–3894. 10.1002/jcp.24101 22552906

[18] Anil N, Singh M. CD4(+)CD25(high) FOXP3(+) regulatory T cells correlate with FEV1 in North Indian children with cystic fibrosis. Immunol Invest. 2014;43: 535–543. 10.3109/08820139.2014.888447 24661230

[19] Bernardi DM, Ribeiro AF, Mazzola TN, Vilela MM, Sgarbieri VC. The impact of cystic fibrosis on the immunologic profile of pediatric patients. J Pediatr (Rio J). 2013;89: 40–47. 10.1016/j.jped.2013.02.007 23544809

[20] Smith DJ, Hill GR, Bell SC, Reid DW. Reduced mucosal associated invariant T-cells are associated with increased disease severity and Pseudomonas aeruginosa infection in cystic fibrosis. PLoS One. 2014;9: e109891 10.1371/journal.pone.0109891 25296025 PMC4190362

[21] Rosenfeld M, Emerson J, Williams-Warren J, Pepe M, Smith A, Montgomery AB, et al Defining a pulmonary exacerbation in cystic fibrosis. J Pediatrics. 2001;139: 359–365. 11562614 10.1067/mpd.2001.117288

[22] Australian Cystic Fibrosis Data Registry. Cystic Fibrosis in Australia 2012. http://www.cysticfibrosis.org.au/media/wysiwyg/CF-Australia/medical-documents/ACFDR_2012/ACFDR_Annual_Report_2012r.pdf

[23] Brett MM, Simmonds EJ, Ghoneim AT, Littlewood JM. The value of serum IgG titres against Pseudomonas aeruginosa in the management of early pseudomonal infection in cystic fibrosis. Arch Dis Child. 1992;67: 1086–1088. 1417051 10.1136/adc.67.9.1086 PMC1793633

[24] Vargas-Rojas MI, Ramirez-Venegas A, Limon-Camacho L, Ochoa L, Hernandez-Zenteno R, Sansores RH. Increase of Th17 cells in peripheral blood of patients with chronic obstructive pulmonary disease. Respir Med. 2011;105: 1648–1654. 10.1016/j.rmed.2011.05.017 21763119

[25] Noack M, Miossec P. Th17 and regulatory T cell balance in autoimmune and inflammatory diseases. Autoimmun Rev. 2014;13: 668–677. 10.1016/j.autrev.2013.12.004 24418308

[26] Hovhannisyan Z, Treatman J, Littman DR, Mayer L. Characterization of interleukin-17-producing regulatory T cells in inflamed intestinal mucosa from patients with inflammatory bowel diseases. Gastroenterology. 2011;140: 957–965. 10.1053/j.gastro.2010.12.002 21147109 PMC3049831

[27] Lee RJ, Foskett JK. Ca(2)(+) signaling and fluid secretion by secretory cells of the airway epithelium. Cell Calcium. 2014;55: 325–336. 10.1016/j.ceca.2014.02.001 24703093

[28] McLoughlin RM, Calatroni A, Visness CM, Wallace PK, Cruikshank WW, Tuzoava M, et al Longitudinal relationship of early life immunomodulatory T cell phenotype and function to development of allergic sensitization in an urban cohort. Clin Exp Allergy. 2012;42: 392–404. 10.1111/j.1365-2222.2011.03882.x 22092655 PMC4162345

[29] Holt PG. Infection and the development of allergic disease. Allergy. 2011;66 Suppl 95: 13–15. 10.1111/j.1398-9995.2011.02623.x 21668843

[30] Dosanjh AK, David E, Robbins RC. The bronchoalveolar lavage fluid of cystic fibrosis lung transplant recipients demonstrates increased interleukin-8 and elastase and decreased IL-10. J Interfer Cytok Res. 1998;18: 851–854. 10.1089/jir.1998.18.851 9809620

[31] Walter GJ, Evans HG, Menon B, Gullick NJ, Kirkham BW, Cope AP, et al Interaction with activated monocytes enhances cytokine expression and suppressive activity of human CD4+CD45ro+CD25+CD127(low) regulatory T cells. Arthritis Rheum. 2013;65: 627–638. 10.1002/art.37832 23280063 PMC3947722

[32] Schadenberg AW, Vastert SJ, Evens FC, Kuis W, van Vught AJ, Jansen NJ, et al FOXP3+ CD4+ Tregs lose suppressive potential but remain anergic during transient inflammation in human. Eur J Immunol. 2011;41: 1132–1142. 10.1002/eji.201040363 21381018

[33] Fazekas de St Groth B, Zhu E, Asad S, Lee L. Flow cytometric detection of human regulatory T cells. Methods Mol Biol. 2011;707: 263–279. 10.1007/978-1-61737-979-6_17 21287341

[34] Gratz IK, Truong HA, Yang SH, Maurano MM, Lee K, Abbas AK, et al Cutting Edge: memory regulatory t cells require IL-7 and not IL-2 for their maintenance in peripheral tissues. J Immunol. 2013;190: 4483–4487. 10.4049/jimmunol.1300212 23543753 PMC3660612

[35] Himmel ME, MacDonald KG, Garcia RV, Steiner TS, Levings MK. Helios+ and Helios- cells coexist within the natural FOXP3+ T regulatory cell subset in humans. J Immunol. 2013;190: 2001–2008. 10.4049/jimmunol.1201379 23359504

