# Supplementary material for: High Peripheral Blood Th17 Percent Associated with Poor Lung Function in Cystic Fibrosis
Source: PLoS One. 2015 Mar 24;10(3):e0120912. doi: 10.1371/journal.pone.0120912 (PMC4372584; doi:10.1371/journal.pone.0120912)
Supplement: S1 Methods — (DOCX) [file pone.0120912.s002.docx]

**Supporting information**

**Methods**

**Blood collection and processing**

Peripheral venous blood samples were collected in lithium heparin and processed within 5 h of specimen collection. Peripheral blood mononuclear cells (PBMC) were isolated from whole blood samples using Histopaque-1077 (Sigma-Aldrich, Castle Hill, NSW, Australia), washed twice with Dulbecco’s phosphate-buffered saline (DPBS)/10% fetal bovine serum (FBS, Bovogen, East Keilor, Vic, Australia) at 300 *g* for 10 minutes at room temperature, then resuspended in 1 mL of DPBS/10% FBS and either cryopreserved for future use or immediately activated and processed for flow cytometry. Preliminary studies comparing responses of fresh and cryopreserved cells from the same sample showed no differences in any of the parameters studied (data not shown).

**Activation, staining and flow cytometric analysis of CD4^+^ cells.**

PBMC were activated and stained according to the instructions of the manufacturer of antibodies and buffers (BD Biosciences, Mountainview, CA). Briefly, 3–4 × 10^6^ PBMC were incubated in one well of a 6-well plate (BD Biosciences) in 4 mL of complete culture medium (CCM; RPMI-1640 [Life Technologies, Mulgrave, VIC, Australia] containing 10% FBS), penicillin and streptomycin (Thermo Fisher). Cells were stimulated with 50 ng/mL of phorbol myristate acetate (PMA; Sigma-Aldrich) and 1 μg/mL of ionomycin (Life Technologies), 2.67 μL of BD GolgiStop (BD Biosciences) was added, and cells were incubated for 5 h at 37 °C in 5% CO_2_ in air.

Following activation, suspended cells were harvested and centrifuged at 250 *g* for 5 minutes in DPBS containing 0.1% (w/v) bovine serum albumin (BSA, Bovogen) and 0.2% (v/v) sodium azide (FACS buffer) then fixed and permeabilized according to the manufacturer’s instructions. PBMCs were washed twice and resuspended in FACS buffer.

For identification of Th17 and Treg cells, 10 μL of the three-colour Human Th17/Treg phenotyping cocktail (BD Biosciences) was added to each cell suspension. For identification of Tr1 and Th3 cells respectively, 10 μL of anti-CD4 PerCP-Cy5.5-conjugated antibody, 2.5 μL of anti-TGF-β1 PE-conjugated antibody, and 1.25 μL of anti-IL-10 APC-conjugated antibody (all BD Biosciences) was added to each cell suspension. For isotype controls, 10 μL of anti-CD4 PerCP-Cy5.5-conjugated antibody, 10 μL of PE-conjugated mouse IgG1κ isotype control and 2.5 μL of APC-conjugated mouse IgG2aκ isotype control (all BD Biosciences) were added. All PBMC samples were then incubated for 40 minutes at room temperature in the dark. Following incubation, 2 mL of FACS buffer was added to each tube, the cells were gently mixed, and washed twice by centrifuging at 500 *g* for 5 min at room temperature. All samples were resuspended in a final volume of approximately 500 μL of FACS buffer, and filtered through 50 μm filter mesh (Sefar, Blacktown, NSW, Australia) into 12 mm × 75 mm tubes (BD Biosciences).

**Flow cytometry data acquisition and analysis**

Cells were analyzed on a 9-colour CyAn ADP flow cytometer (Beckman Coulter, Lane Cove, NSW, Australia) configured with 3 lasers, 11 reporting parameters, and a fixed-alignment quartz cuvette flow cell, using Summit software (version 4.3.2, Beckman Coulter). At least 100 000 gated events were collected for each sample. Postacquisition analysis was performed using FlowJo software (version 9.5.2, Tree Star, Ashland, OR), including autofluorescence correction using a predefined compensation matrix. Representative hierarchal gating schemas are depicted in S3_Fig. CD4^+^ values are expressed as a percent of total lymphocytes; all other subsets are expressed as a percent of CD4^+^ cells.

**S1 Fig. Representative histograms demonstrating the gating strategy for flow cytometric analysis.** All samples were gated on lymphocytes (A) then single cells (B) for determination of CD4+ percent. For determination of CD4^+^ subset percentages, cells were gated on CD4^+^ (C) then subsequent IL-4 vs. IFNγ (D), IL-17 vs. FOXP3 (E) and TGF-β vs. IL-10 (F) histograms were analyzed.

**S2 Fig. Deidentified patient information and results.**
