# Supplementary figures and images for: High Peripheral Blood Th17 Percent Associated with Poor Lung Function in Cystic Fibrosis
Source: PLoS One. 2015 Mar 24;10(3):e0120912. doi: 10.1371/journal.pone.0120912 (PMC4372584; doi:10.1371/journal.pone.0120912)

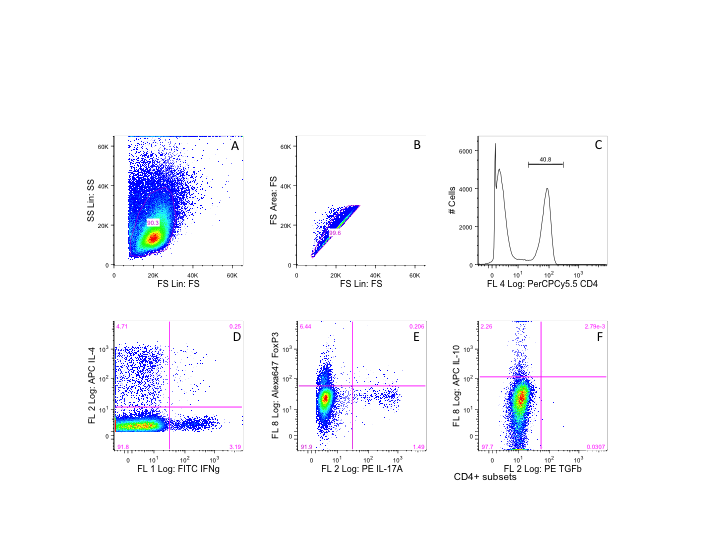

Supplement: S1 Fig — All samples were gated on lymphocytes (A) then single cells (B) for determination of CD4+ percent. For determination of CD4+ subset percentages, cells were gated on CD4+ (C) then subsequent IL-4 vs. IFNγ (D), IL-17 vs. FOXP3 (E) and TGF-β vs. IL-10 (F) histograms were analyzed. (TIFF) [file pone.0120912.s003.tiff]
